# Expression of FAP, ADAM12, WISP1, and SOX11 is heterogeneous in aggressive fibromatosis and spatially relates to the histologic features of tumor activity

**DOI:** 10.1002/cam4.160

**Published:** 2013-11-26

**Authors:** Benjamin S Misemer, Amy P N Skubitz, J Carlos Manivel, Stephen C Schmechel, Edward Y Cheng, Jonathan C Henriksen, Joseph S Koopmeiners, Christopher L Corless, Keith M Skubitz

**Affiliations:** 1 Department of Laboratory Medicine and Pathology, University of Minnesota Medical SchoolMinneapolis, Minnesota; 2 Masonic Cancer Center, University of Minnesota Medical SchoolMinneapolis, Minnesota; 3 Department of Orthopedic Surgery, University of Minnesota Medical SchoolMinneapolis, Minnesota; 4 Division of Biostatistics, University of Minnesota School of Public HealthMinneapolis, Minnesota; 5 Department of Pathology, Oregon Health & Science UniversityPortland, Oregon; 6 Department of Medicine, University of Minnesota Medical SchoolMinneapolis, Minnesota

**Keywords:** ADAM12, desmoids, FAP, fibromatosis, SOX11, WISP1

## Abstract

Aggressive fibromatosis (AF) represents a group of tumors with a variable and unpredictable clinical course, characterized by a monoclonal proliferation of myofibroblastic cells. The optimal treatment for AF remains unclear. Identification and validation of genes whose expression patterns are associated with AF may elucidate biological mechanisms in AF, and aid treatment selection. This study was designed to examine the protein expression by immunohistochemistry (IHC) of four genes, *ADAM12*, *FAP*, *SOX11*, and *WISP1*, that were found in an earlier study to be uniquely overexpressed in AF compared with normal tissues. Digital image analysis was performed to evaluate inter- and intratumor heterogeneity, and correlate protein expression with histologic features, including a histopathologic assessment of tumor activity, defined by nuclear chromatin density ratio (CDR). AF tumors exhibited marked inter- and intratumor histologic heterogeneity. Pathologic assessment of tumor activity and digital assessment of average nuclear size and CDR were all significantly correlated. IHC revealed protein expression of all four genes. IHC staining for ADAM12, FAP, and WISP1 correlated with CDR and was higher, whereas SOX11 staining was lower in tumors with earlier recurrence following excision. All four proteins were expressed, and the regional variation in tumor activity within and among AF cases was demonstrated. A spatial correlation between protein expression and nuclear morphology was observed. IHC also correlated with the probability of recurrence following excision. These proteins may be involved in AF pathogenesis and the corresponding pathways could serve as potential targets of therapy.

## Introduction

Aggressive fibromatosis (AF), also known as desmoid tumor, is characterized by a poorly circumscribed monoclonal proliferation of myofibroblastic cells with variable collagen deposition. AF is locally invasive, but rarely metastasizes, although on occasion it may be multifocal (reviewed in ref. 1,2). Tumors are morphologically heterogeneous. In some areas, the cells have elongated nuclei that stain darkly with hematoxylin, imparting an appearance of “closed”/“transcriptionally inactive” heterochromatin often separated by extensive collagen. In other areas, cells have oval nuclei containing pale-staining, dispersed, or vesicular euchromatin and small nucleoli, imparting an “open”/“transcriptionally active” appearance 3.

The myofibroblastic cells of AF have histologic similarities to the proliferative phase of wound healing, and AF has been associated with trauma, pregnancy, and the use of oral contraceptives (reviewed in ref. 1,2). Several recurrent chromosomal abnormalities have been reported in AF 4–7. Clonal chromosomal changes occur in ∼46% of deep AF and ∼10% of superficial fibromatosis 4.

The Wnt (*β*-catenin) pathway has been strongly implicated in the pathogenesis of AF 1,2,8–10. A mutation in the *β*-catenin gene (*CTNNB1*) 5,6,9,10 occurs in ∼90% of cases. The location of the mutation may influence the clinical course of the disease 5,10. Although most cases are sporadic, AF is about 1000-fold more frequent in patients with the Gardner variant of familial adenomatous polyposis, in which there is a germ-line mutation in the APC gene, which is involved in Wnt signaling and regulates *β*-catenin degradation 5,6,8,9. About 2% of desmoid tumors occur in patients with familial adenomatous polyposis, in whom it is a common cause of death 11.

The clinical course of AF is quite variable, and optimal treatment remains unclear 1,12–16. A variety of medical treatments and radiation therapy are useful in some patients 15,16. Trauma, including surgery, can stimulate AF growth, leading some to discourage surgery as an initial treatment of the disease, especially in the case of familial adenomatous polyposis-associated AF 12–14. Recurrence after surgery is common, with rates up to 40–50% reported 14; however, for tumors amenable to wide resection without significant morbidity in symptomatic patients, surgery remains the preferred approach.

In a previous study, four gene transcripts, *ADAM12*, *FAP, WISP1*, and *SOX11*, were overexpressed in AF compared with 16 different types of nonneoplastic tissue 2. In this study, we examined the expression of these four genes at the protein level using immunohistochemistry (IHC) and digital image analysis to evaluate tissue localization and look for clinical correlations.

## Materials and Methods

### Tissue samples

Twenty-nine formalin-fixed paraffin embedded (FFPE) tissue specimens and annotation data were obtained with University of Minnesota (UMN) Institutional Review Board approval. The tissues included the 12 cases used in gene identification 2, as well as 17 additional cases (Supporting Information).

### Immunohistochemical staining

Five micrometer sections were mounted on TruBond (Tru Scientific Bellingham, WA) slides and stained using primary antibodies to ADAM12, FAP, WISP1, and SOX11 (Supporting Information). Species-matched secondary antibody kits (Vecta Stain ABC, Burlingame, CA) were used with diaminobenzidine (DAB) to detect the primary antibody.

### Digitization

Slides were scanned using a whole-slide imaging system (ScanScope XT, Aperio Technologies, Vista, CA) and annotations were drawn on whole-slide images using a pen tablet screen to exclude areas not representative of AF fibroblasts/myofibroblasts, including large vessels and artifacts such as tissue folds. Resulting annotated areas representative of AF were divided into a grid of 0.25 ×0.25 mm tiled regions of interest (ROIs) using SigMap software previously described 17 (Supporting Information).

### Pathological analysis

All slides and digital images were reviewed in a blinded manner by a pathologist with expertise in the analysis of soft tissue tumors (J. C. M.). A “morphologic tumor activity” (MTA) score, determined by light microscopy, was assigned within each of 322 randomly chosen ROIs, on negative control slides, using a scale of 1 (inactive), 2 (probably inactive), 3 (probably active), and 4 (active).

### Digital analysis

A measurement of stained surface area and stain density within ROIs using Color Deconvolution (Aperio) was performed. This allowed for independent measure of a specific stain in areas with overlap by determining the amount and density of each component color channel required to make up the recorded color of a pixel (Supporting Information). This algorithm reports the percent positive in a region for a given staining component in three light transmission intensity thresholds and the average optical density of that stain 18,19. For hematoxylin, the thresholds were adjusted so that: weak positive detected all hematoxylin-counterstained tissue, medium positive detected pathologist-confirmed “active”/euchromatic-appearing nuclear areas, and strong positive detected “inactive”/heterochromatic-appearing nuclear areas. In addition, the average nuclear size was measured within ROIs on the corresponding negative control slides using IHC Nuclear Count software (Aperio).

Deconvolution of the hematoxylin channel allowed the generation of a nuclear chromatin density ratio (CDR), defined as the ratio of nuclear area with medium-positive hematoxylin staining intensity (corresponding to “active” nuclei) to nuclear area with strong-positive hematoxylin staining intensity (corresponding to “inactive” nuclei). CDR was quantified as a surrogate for cellular activity. The deconvolution of the DAB channel allowed the quantitation of IHC staining for each of the four IHC stains within each ROI, defined by the average optical density of DAB times the percent of area staining positive with DAB.

### β-catenin mutation analysis

Tumor DNA was extracted from FFPE tissue and screened for hotspot mutations in *CTNNB1* using a combination of multiplexed primer extension assays and a mass spectroscopy readout (Sequenom MASSArray system) as previously described 20 (Supporting Information).

### Statistical analysis

The association between pathologic MTA, log of the nuclear size, and log CDR was summarized by Pearson's correlation coefficient. Confidence intervals for the correlation coefficient and *P*-values were calculated using a bootstrap procedure that accounted for potential correlation between measurements on a single individual. A similar analysis was used to evaluate the correlation between log CDR and IHC staining expression of FAP, ADAM12, SOX11, and WISP1. In addition, protein expression and CDR were compared for two groups of samples reported in an earlier study 2 using generalized estimating equations (GEE) to account for potential correlation between samples from the same individual. Finally, a similar GEE analysis was completed to compare IHC staining between subjects based on clinical outcomes.

## Results

### Histologic features of AF specimens

AF samples exhibited striking morphologic intra- and intertumoral heterogeneity. Some tumor areas appeared “inactive,” characterized by sparse cells with narrow, darker staining nuclei, and no mitoses. In general, there was more collagen deposition in regions where the cells appeared inactive. Other areas appeared histologically “active,” characterized by cells with plump, light-staining oval nuclei, greater cell density, and rare mitotic activity.

### Immunohistochemical staining for ADAM12, FAP, SOX11, and WISP1

**Figure 1:**
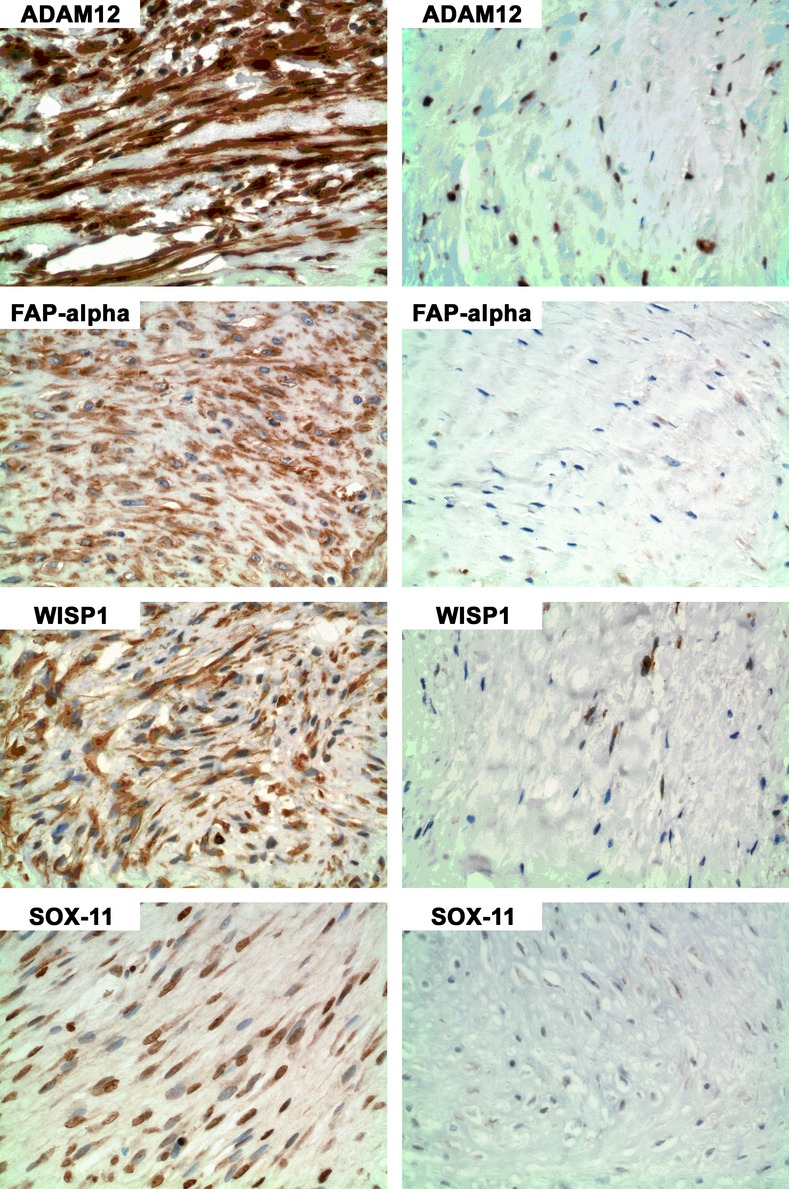
Immunohistochemistry (IHC) for ADAM12, FAP, WISP1, and SOX11 in areas of more “active” cells (left column) and less “active” cells (right column). In regions of “active” cells ADAM12 staining was primarily nuclear and cytoplasmic; FAP staining was primarily cytoplasmic; WISP1 was cytoplasmic; and SOX11 was primarily nuclear.

Each of the four proteins of interest was detected in the AF fibroblastic/myofibroblastic cells by IHC (Fig. 1): ADAM12 (nuclear and cytoplasmic), FAP (cytoplasmic), WISP1 (cytoplasmic), and SOX11 (nuclear). The intensity of staining of all four markers was variable between the different AF samples. ADAM12, SOX11, and WISP1 were also detected in endothelial cells within the AF samples (not shown).

**Figure 2:**
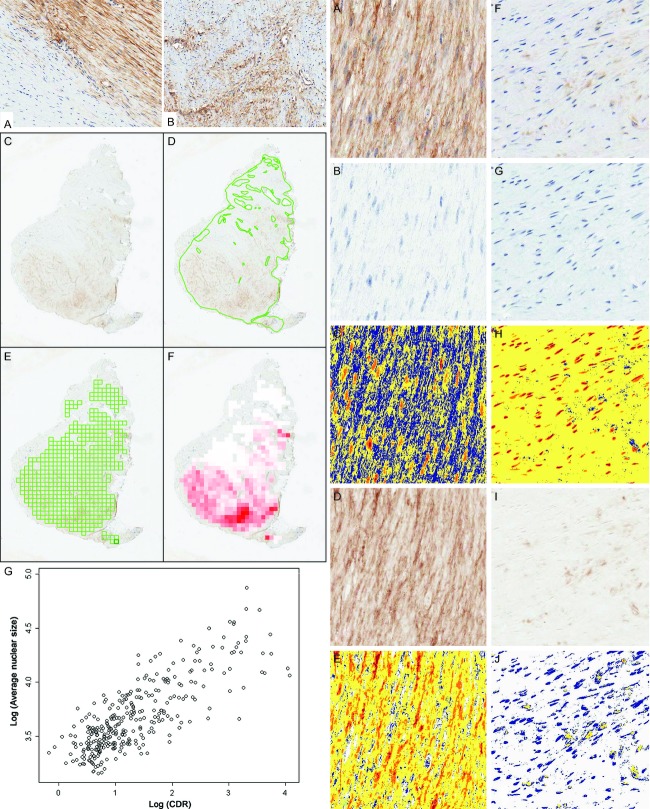
Left panel (A) IHC for FAP demonstrates abrupt transition between “inactive” and negative-staining tumor at left, and “active” tumor with diffuse moderate intensity staining at right. (B) Gradually blended areas of protein expression. *Left panel* (C–F) Representation of image processing used, with FAP staining of AF. Tiled ROIs for each whole-slide image were automatically generated using SigMap software. (C) Scanned slide of DAB stain with H&E counterstain, (D) manual annotations to remove areas not representative of AF including artifacts and vessels, (E) tiled ROI grid overlayed on scanned image, and (F) heat map of DAB staining measurement. The color gradation directly correlates with the amount of stain in the ROI, with increasing intensity of red indicating a higher DAB quantification value. *Left panel* (G) Scatter plot of log [average nuclear size] versus log [CDR]. *Right panel* (A–J) Example of color deconvolution showing representative images of an IHC stain of FAP in areas of more active (A–E) and less active (F–J) AF. Color deconvolution software deconvolved hematoxylin staining (B and G) from DAB staining (D and I) by RGB color components. Staining intensity was separately measured in each color channel. Pseudocolors show: strong intensity threshold (red), medium intensity (orange), weak intensity (yellow), and counterstained tissue not positive (blue), for hematoxylin (C and H) and DAB (E and J). IHC, immunohistochemistry; AF, aggressive fibromatosis; ROI, region of interest; CDR, chromatin density ratio.

In general, the degree of immunostaining appeared higher in “active-appearing” tumor areas (Fig 1, left panels) than in “inactive-appearing” areas (Fig 1, right panels). Marked intratumoral heterogeneity of staining was observed. The demarcation between areas staining positively and negatively by IHC was sometimes abrupt, as seen for FAP staining in Figure 2A, left. However, areas with gradations of staining often blended more gradually (Fig. 2B, left).

### Analysis of heterogeneity

Because both tumor morphology and IHC staining were markedly heterogeneous, we sought to examine quantitatively whether IHC expression was related to morphologic measures of tumor activity. To better quantify morphologic tumor activity (MTA), as determined by nuclear morphology, image analysis was performed. To reduce the problem of spatial heterogeneity each sample was subdivided into ROIs. As described in the Materials and Methods section, whole-slide images were generated for each slide (Fig. 2C, left). Images were manually annotated to exclude areas not representative of AF such as technical artifacts and prominent vessels (Fig. 2D, left), and tiled ROIs were automatically generated within retained tumor areas (Fig. 2E, left). A graphic heat map of DAB staining in the ROI grid is shown in Figure 2F, left, where the red color intensity directly correlates with DAB stain intensity.

### Measurements of immunohistochemical staining, nuclear size, and chromatin density

A nuclear object counting algorithm can be unreliable with intense positive cytoplasmic and nuclear IHC staining. Therefore, a color deconvolution analysis was performed to obtain an estimate of nuclear size in the IHC-stained slides as described in the Methods. Using hematoxylin component data, we defined a digital pathologic surrogate for nuclear size. An example of extracted hematoxylin color deconvolution is shown in Figure 2, right panel. The original FAP IHC data (raw slide images) are shown for an “active” ROI (Fig. 2A, right) and an “inactive” ROI (Fig. 2F, right). Image data were deconvoluted to isolate the hematoxylin component (Fig. 2B and G, right). Nuclear hematoxylin densities are shown in false color images in which orange is medium density (“active” nuclei), which predominates in Figure 2C, right, and red is high density (“inactive” nuclei), which predominates in Figure 2H, right.

From these data, we calculated a CDR defined as the ratio of nuclear area with medium density (active-appearing nuclei) divided by nuclear area with high density (inactive-appearing nuclei). IHC nuclear count (Aperio) was configured to count nuclei and measure nuclear sizes on corresponding negative control slides.

### Correlation of average nuclear size and CDR

Because transcriptionally “active” nuclei have greater surface area on histologic sections, it was expected that CDR would positively correlate with nuclear size. To test this hypothesis, both Color Deconvolution and IHC nuclear count were run on hematoxylin-counterstained slides to generate CDR and nuclear size data, respectively. As expected, the log [CDR] and log [nuclear size] were strongly correlated in negative control slides (0.79; 95% CI: 0.61, 0.87; *P* < 0.001) (Fig. 2G, left), thus demonstrating that CDR is a reasonable surrogate for nuclear size, a commonly used histopathologic feature of cellular activity.

### Correlation of pathologist-assigned MTA score with nuclear size and CDR

**Figure 3:**
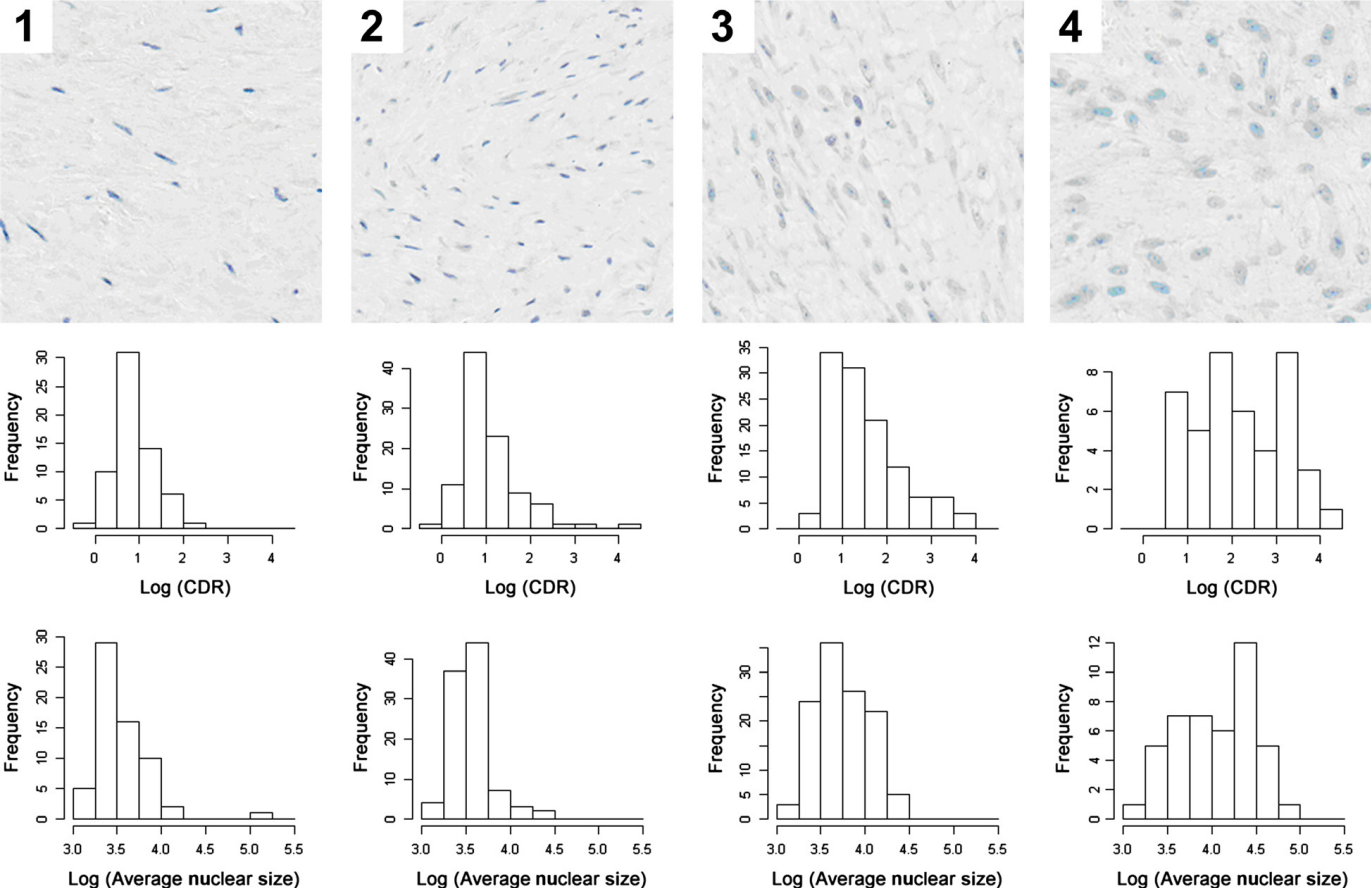
Top row: Representative examples of histologic appearance of MTA scores. MTA score 1 (inactive), MTA score 2 (probably inactive), MTA score 3 (probably active), and MTA score 4 (active). Middle row: Correlation of CDR and MTA. Bottom row: Correlation of nuclear size and MTA score. MTA, morphologic tumor activity; CDR, chromatin density ratio.

To further evaluate disease activity in different regions of tumor, MTA scores (1–4) were assigned by a pathologist as described in Methods, in a blinded fashion for 322 ROI images with matching nuclear size and CDR measurements from hematoxylin-stained slides (Fig. 3, top row).

There was a significant correlation between the MTA score and the log [CDR] (0.46; 95% CI: 0.23, 0.62; *P* < 0.001) (Fig. 3, second row), and between MTA score and log [nuclear size] (0.44; 95% CI: 0.13, 0.64; *P* = 0.010) (Fig. 3, bottom row). These results provide objective evidence that nuclear size and chromatin density contribute to the histologic pattern recognized by pathologists as “active” tumor.

### Correlation of IHC staining with nuclear activity/disease activity

To better define the apparent relationship of the intensity of IHC staining with nuclear properties, color deconvolution was performed to quantify IHC staining intensity in each ROI, as previously described 18,19 (Supporting Information). As illustrated in Figure 2 for representative FAP IHC-stained “active” (Fig. 2A, right) and “inactive” (Fig. 2F, right) ROIs, deconvoluted DAB staining (Fig. 2D and I, right) was quantified. Pseudocolor images use red as high-density DAB staining, orange as moderate, and yellow as low-density DAB staining. Higher staining densities were seen in “active” areas (Fig. 2E, right) compared to “inactive” areas (Fig. 2J, right).

A relationship was found between higher CDR and more intense IHC staining for each of the four antibodies (Supporting Information). There were moderate correlations between the intensity of FAP staining and log [CDR] (*r* = 0.44; 95% CI: 0.27, 0.60; *P* < 0.001), between ADAM12 staining and log [CDR] (*r* = 0.30; 95% CI: 0.25, 0.39; *P* < 0.001), and between WISP1 staining and log [CDR] (*r* = 0.27; 95% CI: 0.10, 0.42; *P* < 0.001). No significant correlation was observed between log [CDR] and SOX11 staining (*r* = 0.14; 95% CI: −0.09, 0.30; *P* = 0.24).

### β-catenin mutation state

β-catenin mutations were found in 11 of the 12 samples of group 1 (the samples studied in the earlier gene expression study 2) (Supporting Information). In Set A two had an S45F mutation, two a T41A mutation, and one was wild type. In Set B, four had an S45F, two an S45P, and one a T41A mutation.

### Correlation of IHC staining with prior gene expression analyses

A significant difference in IHC staining for WISP1, quantified by image analysis, was observed between Set A 2 and Set B (*P* = 0.008). However, no significant difference in IHC staining for FAP, ADAM12, and SOX11 was observed, in contrast to differences in mRNA expression observed in an earlier study 2. No difference in log [CDR] was observed between Sets A and B (not shown).

### Correlation with clinical course of disease

To correlate IHC staining with the clinical outcome in the cases for which clinical follow-up was available, we created the operational definition of “did not recur within 5 years from excision” (six cases) and “recurred within 1 year from excision” (seven cases). Image analysis revealed that there was a statistically significant difference between the groups in IHC staining intensity for FAP and SOX11, but not ADAM12 or WISP1, although a trend was observed (*P* = 0.02, 0.03, 0.06, and 0.09, respectively) with more staining of ADAM12, FAP, and WISP1 and less staining of SOX11 in the subset that recurred by 1 year. No difference in CDR was observed between the two subsets.

## Discussion

This report confirms protein expression of four genes (ADAM12, FAP, WISP1, and SOX11) previously reported to be selectively overexpressed in AF 2. Marked heterogeneity of the expression of these four proteins was found within and between tumor samples. Cellular morphology also varied widely within the tumor samples, with some areas resembling fibroblasts of inactive fibrous tissue, and other areas resembling the active fibroblasts of wound healing. Visual analysis suggested a relationship between nuclear features and IHC staining. Digital image analysis provided objective evidence supporting these initial observations, and also demonstrated a relationship between expression of these four proteins and time to recurrence. In 13 patients with clinical follow-up information, we found that high expression of ADAM12 (*P* = 0.06), FAP (*P* = 0.02), and WISP1 (*P* = 0.09), and low expression of SOX11 (*P* = 0.03), correlated with worse clinical outcome, defined by early (≤1 year) recurrence after excision in seven patients, versus nonrecurrence at ≥5 years in six patients.

Image analysis revealed that the expression of ADAM12, FAP, and WISP1 correlated with the CDR, and thus with the degree of histologic disease activity, whereas a correlation with SOX11 did not reach statistical significance. One possible explanation of the lack of significance observed with SOX11 could be related to its cellular distribution and our methods of DAB quantification. Expression was quantified by combining DAB staining intensity and area. SOX11 was unique among the markers as it was expressed almost exclusively in the nucleus. When cells assume an “active” morphology, the area of the nucleus may expand proportionally less than that of the cytoplasm. Expansion of the cytoplasm provides a larger factor of expansion of expression quantification than the corresponding increase in nuclear size. Therefore, for nuclear stains including area in its assessment may hinder identification of a difference.

The previous gene expression study suggested the existence of two distinct subgroups 2. This study confirmed differences between the two groups in protein expression of WISP1 but not the other three proteins. The inability to detect subsets in this study could reflect the small size of the samples examined (one 5 micron slice), and the regional variability in disease activity.

Mutations in CTNNB1 occur in most AF cases, with the vast majority being one of three point mutations: S45F, T41A, and S45 5,6,8–10. Studies have suggested that the site of the CTNNB1 mutation is associated with differences in clinical behavior of the tumor 6,10,14. In this study, IHC staining with the four antibodies, and tumor “activity,” did not correlate with β-catenin mutation, and there was no clear difference in CTNNB1 mutation between Sets A and B of the earlier gene expression study, although a larger study might identify a correlation.

ADAM12 has been implicated in cell–cell and cell–matrix interactions, may activate or inactivate various signaling molecules, and regulate integrin interaction and cell differentiation 21,22. ADAM12 expression has been demonstrated in a number of malignant diseases 23, and also Dupuytren's disease 24.

FAP, a serine protease with both gelatinase and collagenase activity, is localized to the cell surface/membrane and to the cytoplasm of cells. FAP expression has been detected in tumor stroma, and several different fibrotic diseases, such as idiopathic pulmonary fibrosis and cirrhosis 25. FAP is transiently expressed in some fetal mesenchymal cells, but absent from most normal adult tissues 26. The role of FAP in malignancy is complicated and unclear.

WISP1 is a secreted protein that associates with the extracellular matrix and acts as a growth factor that can regulate diverse cellular functions 27. WISP-1 expression has been reported in a wide array of tumors, notably neurofibromas 28, the desmoplastic tumor stroma from cancers of epithelial origin 29, and AF 2,30,31, and Wnt signaling has been implicated in AF 2,8–10. WISP1 is one of a panel of genes in an expression signature that can distinguish AF from nodular fasciitis 30.

The expression of the nuclear transcription factor SOX11 is temporally regulated throughout development. Most adult tissues do not express SOX11. A recent study found much higher SOX11 expression in mesenchymal stem cell lines than in differentiated mesenchymal cells such as fibroblasts and adipocytes 32. Deregulation of SOX11 expression has been shown to be common to many neoplasms 33, and SOX11 was found to be overexpressed in liposarcomas and Wilms tumors compared with normal tissues and several other malignant tumors 34.

Studies suggest an important role of both ADAM12 and WISP1 in pulmonary fibrosis 35. ADAM12 is expressed in diseases associated with fibrosis, and may be involved in signaling of epidermal growth factor (EGF), insulin like growth factor (IGF), and transforming growth factor, beta1 (TGFB1) (reviewed in ref. 35). WISP1 is upregulated in idiopathic pulmonary fibrosis 36, and recombinant WISP1 enhances extracellular matrix deposition by fibroblasts 36. WISP1 is also upregulated in a mouse model of bleomycin-induced lung injury, and antibody to WISP1 attenuates fibrosis in this model 36.

Thus, a possible model of AF pathogenesis is an activating stimulus in the setting of a deregulation of β-catenin, leading to upregulation of β-catenin. β-catenin then binds the WISP1 promoter and increases WISP1 production. In addition, WISP1 induces β-catenin (prosurvival signal) nuclear translocation 37, further promoting WISP1 production. WISP1 then binds its receptor and recruits profibrotic ADAM12-positive cells from a PDGFR-positive precursor pool. These recruited cells are FAP positive, and produce various ECM molecules leading to fibrosis. The role of SOX11 in this process remains unclear, although other studies have shown that it aides in mesenchymal stem cell proliferation and pluripotent potential retention. In many cases, these cells may regress or become less active, with a concomitant decrease in expression of FAP, ADAM12, WISP1, and SOX11, and stabilization or regression of the clinical AF disease activity. The fact that AF can regress or stabilize implies that the cells in these cases are still in some way sensitive to a regulatory system similar to that of wound healing, yet remain susceptible to reactivation by unknown, possibly multiple, proinflammatory and/or profibrotic stimuli that may be induced by injury or other physiological conditions such as pregnancy. Mouse models of bleomycin lung toxicity in which antibodies to WISP1 inhibit fibrosis suggest that WISP1 may be a particularly attractive potential therapeutic target in AF. The response of AF to certain chemotherapy drugs, and the potential similarity of the process, suggests that consideration be given to trials of these drugs in other serious fibrotic diseases, such as aggressive forms of interstitial pulmonary fibrosis.

Studies of AF may be informative in other malignancies as well. Tumors have been described as “wounds that do not heal” 38 and AF in many ways resembles wound healing. As the tumor stroma in desmoplastic reactions is composed of a more uniform and nontransformed population of cells, it provides an interesting therapeutic target. AF may provide clues to the potential role of tumor stroma in cancer biology 39.

Thus, the results confirm, on the protein expression level, earlier findings of overexpression of these genes in microarray studies, and demonstrate the regional variation in tumor activity within and among AF cases. The expression of these proteins correlated with tumor areas in which cells had more “active” appearing nuclear features, and high expression of ADAM12, FAP, and WISP1, and low expression of SOX11, correlated with worse clinical outcome. The study also demonstrated that the CDR is a viable surrogate for assessing nuclear size, and that digital imaging may detect prognostic information not be readily apparent by visual inspection.

## Supporting Information

Figure S1 Comparison of IHC quantification for each of the four IHC stains with log (CDR).

Table S1 Sample characteristics.
