# Supplementary figures and images for: Expression of FAP, ADAM12, WISP1, and SOX11 is heterogeneous in aggressive fibromatosis and spatially relates to the histologic features of tumor activity
Source: Cancer Med. 2013 Nov 26;3(1):81–90. doi: 10.1002/cam4.160 (PMC3930392; doi:10.1002/cam4.160)

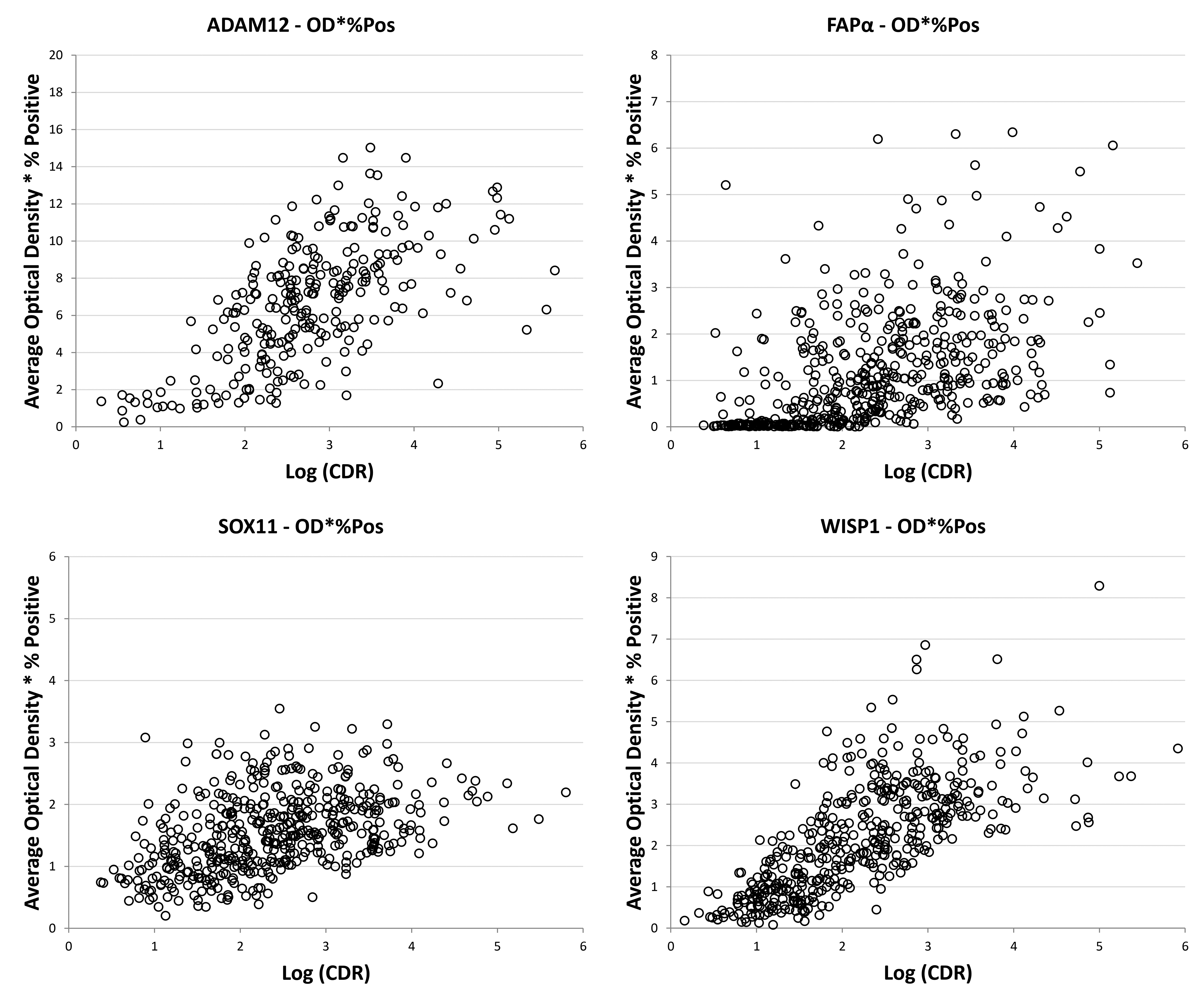

Supplement: Figure S1 — Comparison of IHC quantification for each of the four IHC stains with log (CDR). [file cam40003-0081-sd1.tif]
