# Supplementary material for: Expression of FAP, ADAM12, WISP1, and SOX11 is heterogeneous in aggressive fibromatosis and spatially relates to the histologic features of tumor activity
Source: Cancer Med. 2013 Nov 26;3(1):81–90. doi: 10.1002/cam4.160 (PMC3930392; doi:10.1002/cam4.160)
Supplement: Table S1 — Sample characteristics. [file cam40003-0081-sd2.doc]

**Time to Time to Beta**

**recurrence recurrence catenin**

**Sample Age Gender Site after excision (years) Margins mutation**

AF-1 68 F neck no follow-up at 5 years WT

AF-2 23 M shoulder no follow-up at 5 years S45F

AF-3 35 F rectus sheath no follow-up at 5 years S45F

AF-4 13 M buttock NED at 18 months T41A

AF-5 36 M thigh no follow-up at 4 years T41A

AF-6 21 M sacral recurred at 1 year, chemotherapy S45P

excised, recurred at 6 months

AF-7 22 F buttock recurred at 1 year, chemotherapy S45F

excised, XRT, recurred at 3 years,

reexcised

AF-8 33 F rectus sheath NED at 3 years S45F

AF-9 33 F rectus sheath no follow-up at 4 years S45F

AF-10 23 M pelvis no follow-up at 4 years S45P

AF-11 19 M flank NED at 29 months S45F

AF-12 16 M arm no follow-up at 4 years T41A

AF-13 23 F hip no recurrence at 9 years >5 Positive

AF-14 62 M arm no recurrence at 7.5 years >5 Positive

AF-15 36 F thigh no recurrence at 9 years >5 Positive

AF-16 34 F knee recurred at 14 years >5 Positive

AF-17 43 M thigh no recurrence at 5 years >5 Positive

AF-18 57 M knee no recurrence at 11 years >5 Negative

AF-19 16 F calf 0.8 years </=1 Positive

AF-20 25 F hip 0.7 years </=1 Positive

AF-21 10 M arm 0.4 years </=1 Negative

AF-22 21 F hip 1 year </=1 Negative

AF-23 42 M shoulder 1 year </=1 Negative

AF-24 23 M thigh 0.6 year </=1 Positive

AF-25 27 F buttock 1 year </=1 Indeterminate^a^

AF-26 80 F shoulder recurred at 5 years Positive

AF-27 36 F right chest wall no progression at 3 years

AF-28 23 M mesentery – no surgery treated with chemotherapy

Gardner syndrome

AF-29 30 M back and mesentery no surgery treated with chemotherapy

Gardner syndrome chemotherapy

^a^fragmented
