# Supplementary material for: Expression of FAP, ADAM12, WISP1, and SOX11 is heterogeneous in aggressive fibromatosis and spatially relates to the histologic features of tumor activity
Source: Cancer Med. 2013 Nov 26;3(1):81–90. doi: 10.1002/cam4.160 (PMC3930392; doi:10.1002/cam4.160)
Supplement: Supplementary file 3 [file cam40003-0081-sd3.docx]

**Supplemental materials**

**Materials and Methods**

**Immunohistochemical Staining**

Five um sections were cut with a microtome (Leica Microsystems, Bonnackburn, IL), placed in a water bath heated to 35°C, and mounted on TruBond 380 slides (Tru Scientific Bellingham, WA). Slides were dried overnight at room temperature and heated to 60°C for 15 minutes before staining. Samples were de-paraffinized in pure xylene, and then rehydrated through a series of graded ethanol washes. Endogenous peroxidase activity was quenched with 3% hydrogen peroxide in phosphate buffered saline. Non-specific antibody staining was blocked by treatment for one hour with a solution of Background Sniper (Biocare Concord, CA) for ADAM12, FAP, and WISP1, and with both Background Sniper and 5% w/v dried non-fat milk (Roundy’s, Milwaukee WI) for SOX11. Primary antibodies were mixed with diluted blocking solution (1:10 with PBS) for final concentrations used in overnight 4°C incubations on tissue sections as follows: 1.4 ug/ml rabbit polyclonal anti-human ADAM12 (Proteintech Group Chicago, IL), 2 ug/ml rat monoclonal D8 anti-human FAP (Vitatex Stony Brook, NY), 0.5 ug/ml rabbit polyclonal anti-human SOX11 (Santa Cruz Biotechnology, Santa Cruz, CA), 4 ug/ml purified mouse polyclonal anti-human WISP-1 (Abnova, Taipei City, Taiwan). Bound primary antibody was detected using a species-appropriate secondary antibody kit (Vecta Stain ABC, Burlingame, CA), and 3,3,-diaminobenzidine (DAB) chromagen (Covance Princeton, NJ). Samples were then counter-stained with Harris Hemotoxylin (Surgi-Path, Richmond, IL) and cover slips were sealed with mounting medium (Permount, Thermo Fisher Scientific, Waltham, MA). Corresponding negative controls slides were generated using species- and isotype-matched pre-immunized immunoglobulin. Positive control sections from tissues known to express the target protein were run with each experiment as follows: ADAM12: ductal breast cancer, FAP: pancreatic cancer, SOX11: placenta, and WISP1: ileum.

**Digitization/Region of Interest (ROI) Devleopment**

Slides were scanned using a whole slide imaging system (ScanScope XT, Aperio Technologies, Vista, CA) fitted with a 20x/0.75 Plan Apo objective lens (Olympus, Center Valley, PA) at a resolution of 0.5µm/pixel. Images were saved and retrieved on a server using whole slide file information management software (Spectrum 11.2, Aperio). Using a pen tablet screen (Cintiq 21UX, Wacom, Kazo-shi, Saitama, Japan) annotations were drawn on whole slide images viewed at high resolution using annotation software (ImageScope 11.2, Aperio). These annotations were made to include tumor boundaries and, within tumor areas, to exclude areas not representative of AF fibroblasts/myofibroblasts (including large vessels) and artifacts such as tissue folds. Within resulting annotated areas representative of AF, annotations were divided into a grid of 0.25 x 0.25 mm tiled annotation regions of interest (ROIs) using SigMap software previously described (21).

**Digital Analysis**

Within ROIs, up to two measurement tools were used to generate data in five variables. The surface area and density of DAB and hematoxoylin stains were measured using Color Deconvolution (Aperio). The color deconvolution algorithm splits the image into separate color channels for each stain used by determining the amount and density of each stain component required to make up the color of an individual pixel, and allows for independent measurements of a selected stain channel. The color deconvolution image analysis algorithm was calibrated for the color vectors of the two stains used (blue, hematoxylin; and brown, DAB): representative areas where only one stain was present were measured and their average RGB optical densities were entered into the algorithm for each stain. The calibrated algorithm reports the percent positive in a region for a given staining component in three light transmission intensity thresholds and the average optical density of that stain. Using the Beer-Lambert law the amount of stain present is proportional to the degree of reduction of light passing through the specimen (22,23), measured by optical density (absorbance).

Intensity thresholds were adjusted for each stain. For hematoxylin, the thresholds for weak positive intensity blue were adjusted to detect all hematoxylin-counterstained tissue areas present, medium positive intensity blue was adjusted to detect pathologist-confirmed open/”active”/euchromatic-appearing nuclear areas, and strong intensity blue

was adjusted to detect pathologist-confirmed closed/”inactive”/heterochromatic-appearing nuclear areas. The calibrated color deconvolution algorithms were run twice to measure the hematoxylin and DAB staining separately.

The measurement tool IHC Nuclear Count (Aperio) collected data on average nuclear size within ROIs on corresponding negative control slides of the same tissue. All experimental slides had color deconvolution run for both DAB and hematoxylin channels. Hematoxylin color deconvolution and nuclear count were run on all negative control slides. The values returned by deconvolution of the hemotoxylin channel allowed for the generation of a nuclear chromatin density ratio (CDR) defined as the ratio of nuclear area with medium positive hematoxylin staining intensity (corresponding to open/active nuclei), to nuclear area with strong positive hematoxylin staining intensity (corresponding to closed/inactive nuclei in the respective ROI). CDR was quantified as a surrogate for cellular activity. The values returned by deconvolution of the hematoxylin channel allowed for the generation of IHC staining scores for each of the four IHC stains were quantified within each ROI as defined by the average optical density of DAB times the percent of area staining positive in the DAB color vector within tissue areas (the sum of all image areas exceeding weak, medium, and strong staining intensity thresholds).

**Correlation of IHC staining with nuclear activity/disease activity**

As described above, variable but positive immunostaining for all 4 antibodies was noted in the AF samples, and visual review of the slides suggested more prominent staining in areas where cellular morphology suggested more tumor activity (higher MTA scores), and less intense staining in areas with lower MTA scores. To better define the apparent relationship of the intensity of immunostaining with the 4 antibodies of interest with nuclear properties, color deconvolution was performed to isolate the brown/DAB component. IHC staining intensity in each ROI was quantified as the product of average optical density (OD) multiplied by the percentage of tissue area exceeding a minimal brown threshold (OD*%Pos) times total stained area, yielding ODmm^2^ = average stain density times surface area stained, as previously described (22,23). OD is linearly related to the amount of staining present (22), and we previously found that the metric OD*%Positive produces highly similar data to visual evaluation by a pathologist (23).

**Beta catenin mutation analysis**

Tumor DNA was extracted from FFPE tissue and screened for hotspot mutations in *CTNNB1* using a combination of multiplexed primer extension assays and a mass spectroscopy readout (Sequenom MASSArray system) exactly as previously described (24). The assays cover 21 different possible single nucleotide substitutions affecting codons 32, 33, 34, 37, 41 and 45, which together account for approximately 79% of all *CTNNB1* reported in the COSMIC database. All mutations identified on the MASSArray were confirmed by standard, bi-directional Sanger sequencing on an ABI 3130 sequencer.

**Supplemental Table 1**. Sample characteristics.

**Supplemental Figure 1**. Comparison of IHC quantification for each of the four IHC sains with log (CDR).
